# Laboratory and Numerical Analysis of Steel Cold-Formed Sigma Beams Retrofitted by Bonded CFRP Tapes—Extended Research

**DOI:** 10.3390/ma13214960

**Published:** 2020-11-04

**Authors:** Ilona Szewczak, Katarzyna Rzeszut, Patryk Rozylo, Malgorzata Snela

**Affiliations:** 1 Faculty of Civil Engineering, Lublin University of Technology, 36 Nadbystrzycka Str, 20-618 Lublin, Poland; m.snela@pollub.pl; 2 Faculty of Civil and Transport Engineering, Institute of Building Engineering, Poznan University of Technology, 5 Marii Skłodowskiej-Curie Str, 60-965 Poznań, Poland; katarzyna.rzeszut@put.poznan.pl; 3 Faculty of Mechanical Engineering, Lublin University of Technology, 36 Nadbystrzycka Str, 20-618 Lublin, Poland; p.rozylo@pollub.pl

**Keywords:** numerical analysis, thin-walled cold-formed steel beam, reinforcement method, composite tapes, adhesive

## Abstract

The presented research is a part of a broader study of strengthening methods closely associated with cold-formed sigma steel beams with tapes made of Carbon Fiber Reinforcement Polymer/Plastic (CFRP). The presented results are a continuation and extension of the tests described in previous work by the authors and refer to high-slenderness thin-walled steel sigma beams subjected to a significant large rotation. The main idea of this expanded study was to identify the effectiveness of CFRP tapes with respect to different locations, namely at a bottom-tensioned or upper-compressed flange. Six beams with a cross-section of an Σ140 × 70 × 2.5 profile by “Blachy Pruszyński” and made of S350GD steel with a span of L = 270 cm were tested in the four-point bending scheme. Two beams, taken as reference, were tested without reinforcement. The remaining beams were reinforced with the use of a 50-mm wide and 1.2-mm thick Sika CarboDur S512 CFRP tape, with two beams reinforced by placing the tape on the upper flange and two with tape located on the bottom flange. The CFRP tape was bonded directly to the beams (by SikaDur^®^-30 adhesive). Laboratory tests were aimed at determining the impact of the use of composite tapes on the limitation of displacements and deformations of thin-walled structures. In order to perform a precise measurement of displacement, which is, in the case of beams subjected to large rotations, a very difficult issue in itself, the Tritop system and two coupled lenses of the Aramis system were used. Electrofusion strain gauges were used to measure the deformation. In the next step, numerical models of the analyzed beams were developed in the Abaqus program. Good compliance of the results of laboratory tests and numerical analyses was achieved. The obtained results confirm the beneficial effect of the use of tapes (CFRP) on the reduction in displacements and deformations of steel cold-formed elements.

## 1. Introduction

Designers of steel structures constantly strive to design structures that are economical, safe and quick to build up. These requirements are satisfied by cold-formed, thin-walled steel structural elements, and therefore they are used more and more often [1]. With reference to Vlasov’s definition, a bar can be contemplated as thin-walled if the wall thickness is approximately eight times smaller than the longest distance which is registered along the center line (between two extreme points located on the bar cross-section contour), and this is approximately eight times smaller than the bar length. Detailed guidelines concerning the limit values of transverse dimensions for thin-walled elements are indicated in the works [2,3,4].

According to [5], the development of steel thin-walled structures contributes to the reduction in steel consumption up to 50% compared to traditional constructions made of hot-formed sections, assembly time up to 60% and construction costs up to 25%. Due to the increasing use of cold-formed steel elements, it may become more and more necessary to strengthen them—e.g., in the case of increased variable actions. Due to the small cross-sectional thickness of these elements, the possibility of welding and the use of mechanical fasteners is limited. Performing reinforcements by using adhesives may constitute a solution. Composite tapes Carbon Fiber Reinforcement Polymers/Plastics (CFRPs) have been successfully used for years to strengthen concrete, reinforce concrete and masonry structures and, recently, research has even been carried out concerning their use when renewing wooden structures [6].

Composite materials (Fiber Reinforcement Polymers/Plastics (FRPs)) are materials based on high-strength non-metallic fibers, embedded in an epoxy matrix [7]. Manufacturers of composite materials declare that CFRP tapes can be used wherever they are required to strengthen the existing structure due to increased imposed loads (e.g., due to a change in the purpose of the object) or the emergence of new loads resulting, for example, from installing heavy devices [8].

The main properties of CFRP composites depend mainly on the type and direction of the arrangement of carbon fibers, the parameters of the epoxy (resin) used and its volumetric share in the final material, and the drying conditions [9]. On the basis of research [8], the basic advantages of CFRPs include: a more than ten times greater tensile strength along the fibers when compared to conventional structural steel, very high fatigue strength, high durability caused by high resistance to aggressive factors, corrosion resistance and no need for maintenance, low weight and low cross-section. The main disadvantages of CFRP tapes are the anisotropic strength properties (the stiffness and strength of the tapes along the fibers are very high, while in the direction perpendicular to the fibers, these parameters are much weaker [10]), low compressive strength (approximately 10% of the tensile strength) and even lower interlayer shear (debonding) [11], and no resistance to high temperatures due to the presence of epoxy resins [7]. Externally bonded FRP sheets are extensively presented in scientific publications and represent a current issue [12]. The application of FRP structures are not reviewed properly. Recently, FRP materials are applied for column jacketing [13].

Despite the disadvantages, the possibility of obtaining a quick (due to the installation time) and effective increase in the strength parameters of the structure with a relatively low cost contributes to a significant increase in the use of these materials. However, there is still a shortage of published scientific studies that would enable the development of guidelines for the reinforcement of steel thin-walled structures. While the literature includes studies concerning the CFRP reinforcement of cold-formed steel compression elements with circular- [14], channel- [15,16] or hat-shaped sections [17], it is very difficult to find research on beams. The few available include tests of CFRP-reinforced, thin-walled steel T-beams subjected to bending, described in [18], and yet cold-formed steel elements are used, for example, to create purlins in steel halls and (in two-branch systems) as a steel girders. In response to inquiries from the industry concerning the possibility of reinforcing thin-walled steel purlins made of sigma profile, the authors of this paper began laboratory tests with reference to the reinforcement of cold-formed sigma steel beams with composite tapes. The first stage of the research, which confirmed the beneficial effect of using CFRP tapes on the limitation of deformations and displacements of beams, is presented in [1]. This paper describes the next stage of research concerning the strengthening of bent thin-walled steel sigma beams with a lower cross-section and thus greater member slenderness ratio.

The essence of the extension of research results, in the context of the original work [1], is the possibility of carrying out analogous tests for beams of the same length but with a different cross-sectional height, and therefore a different slenderness. In the previous study [1], the behavior of the structure was presented in the context of local and distortional buckling caused by the bending of a high cross-section of the sigma beam (200 × 70 × 2 mm), whereas the studies presented in this paper concern the element with a low cross-section (140 × 70 × 2.5 mm) characterized by the global bucking mode in the form of a large rotation. In the first stage of the studies [1], a good qualitative and quantitative correlation was obtained between the numerical and experimental results; the aim of these studies was also to obtain satisfactory results despite the change of geometric parameters of the structure cross-section. Moreover, the authors set themselves a goal to check whether the influence of CFRP tapes on the structural behavior for the low cross-section sigma beams (characterized by a global form of deformation) will be as significant as in the case of high cross-section sigma beams [1].

## 2. Laboratory Tests

**Figure 1:**
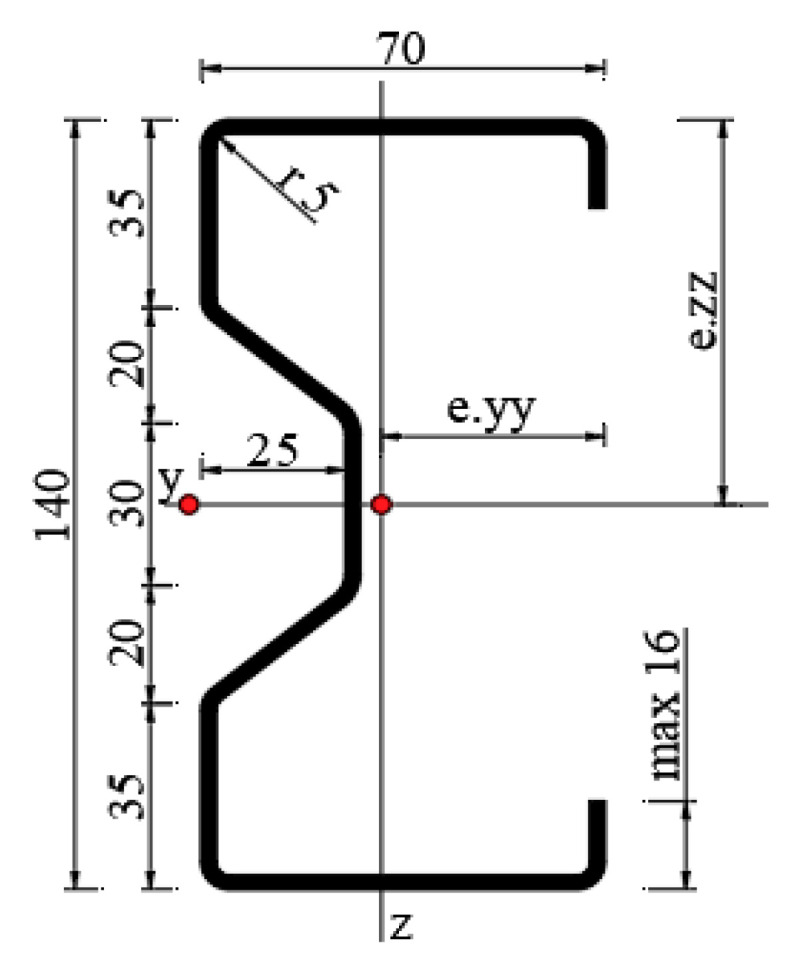
The Σ140 × 70 × 2.5 cross-section geometry.

Six thin-walled steel beams with a section of Σ140 × 70 × 2.5 [19], a length of 300 cm and made of S350GD steel, were included in the study. The geometrical characteristics of the cross-section are given in Figure 1 and in Table 1.

In the table above, FA is the cross-sectional area, e_yy_—the distance of the profile center of gravity with respect to the *z* axis, e_zz_—the distance of the profile center of with respect to the *y* axis, J_y_—the moment of inertia of the section with respect to the *y* axis, J_z_—the moment of inertia of the section with respect to the *z* axis, W_y_—second modulus of area with respect to the *y* axis (minimum), W_z_—second modulus of area with respect to the *z* axis (minimum), i_y_—radius of gyration with respect to the *y* axis, and i_z_—radius of gyration with respect to the *z* axis.

**Figure 2:**
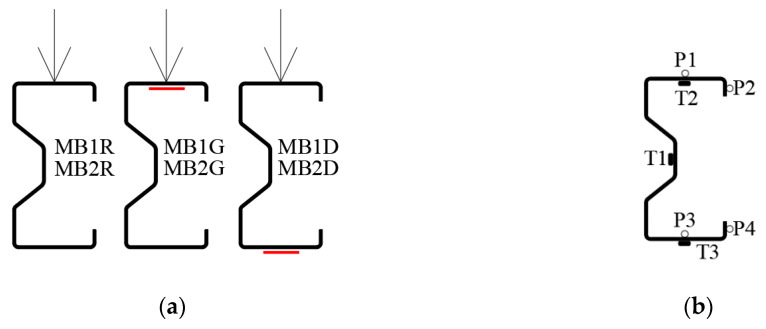
Tested beams: (**a**) sample symbols and Carbon Fiber Reinforcement Polymer/Plastic (CFRP) tapes location; (**b**) measuring points—electrofusion strain gauges (T1, T2, T3) and displacement measurement points (P1, P2, P3, P4), located at the very center of the beam span.

Four of the tested beams were reinforced with CFRP tapes glued to different surfaces: two beams (MB1D, MB2D) were reinforced with CFRP tape located on the outer plane of the bottom flange, whereas two beams (MB1G, MB2G) had reinforcement placed on the upper flange (on the inner plane). Two beams (MB1R, MB2R—reference beams) were also tested without reinforcement. The arrangement of the tapes on the beams section is shown in Figure 2a).

Laboratory tests were carried out to determine the strength parameters of steel. The research was carried out using five samples cut out from the tested thin-walled profiles. The size and shape of the samples resulted from the guidelines of PN-EN ISO 6892-1: 2009 [20]. The biaxial extensometer was used to measure the longitudinal and transverse deformation of the samples. On the basis of conducted tests, the steel yield point was determined (fy = 418.5 MPa), as well as Young’s modulus (E = 201.8 GPa) and Poisson’s ratio (ν = 0.28.) The tested beams were reinforced with Sika CarboDurS carbon fiber strips (CFRP), 50-mm wide and 1.2-mm thick. The CFRP tapes applied on the beams were composed of a unidirectional arrangement of carbon fibers sunk in an epoxy matrix and are characterized by an anisotropic structure. The conducted research allowed to determine the Poisson’s ratio ν = 0.308 and the Young’s modulus E = 142 GPa of the tape used. More details on the parameters of the composite tape are described in [21]. To connect the thin-walled steel beams with the CFRP tape, specialized SikaDur^®^-30 adhesive was used. The most important features of this adhesive include the modulus of elasticity in compression equal to 9600 MPa, minimum compressive strength (75 MPa after 7 days), minimum tensile strength (26 MPa after 7 days), steel deboning (21 MPa after 7 days), shear strength (minimum 16 MPa) and 0.04% shrinkage.

The first stage of laboratory work was the preparation of samples for testing. Before laboratory tests, all beams were degreased and matted with medium grain sandpaper and cleaned in the areas where composite tapes were planned to be applied. The beams were then reinforced by gluing the CFRP tapes. In the study, 207.5-cm long tapes were used, with an effective anchorage length of 30 cm. The effective anchorage length was determined as described in [1]. The tapes were glued to the beams with 1.3-mm thick adhesive. The thickness of the adhesive layer was based on studies conducted by Kowal [22].

At the time of the laboratory tests, the strain was measured using three electrofusion strain gauges (TENMEX TFs-10, resistance 120 Ω ± 0.2%, Lodz, Poland). All electrofusion strain gauges (T1, T2, T3) were located in the middle of the span of each of the tested beams. Figure 2b shows the arrangement of strain gauges on the beam cross-section. Optical measuring systems were also used during the tests. They enable accurate measurement of the displacement of test samples undergoing large rotations. In this study, a special arrangement of two Aramis measuring lenses (GOM, GmbH, Braunschweig, Germany) and a Tritop machine (GOM, GmbH, Braunschweig, Germany) was used. Before the tests, white chalk was applied to the surface of the beams to make it extra matt so that it would not reflect light. Appropriate measuring points with a diameter of 5 mm were glued to the middle of the beam span. The point symbols are shown in Figure 2b. The Tritop system enables the assessment of initial geometric imperfections. The measurement points also enable the generation of one reference coordinate system for two coupled devices in the Aramis system [23]. They were also used for displacements analyses of the tested beams in the GOM Correlate program (GOM, GmbH, Braunschweig, Germany). The method and scope of the application of the Tritop and Aramis systems are discussed in detail in [23].

**Figure 3:**
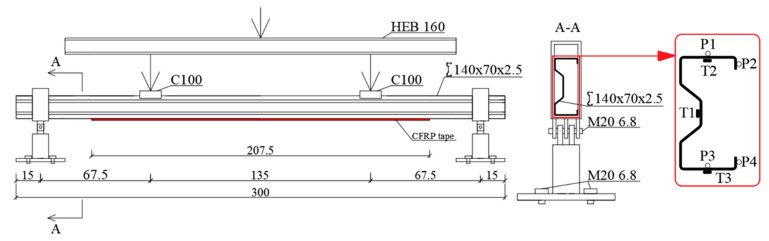
Laboratory stand scheme.

A four-point bending test was carried out using a test stand developed for this purpose. The assumed support spacing was 270 cm and load spacing was 135 cm (Figure 3).

**Figure 4:**
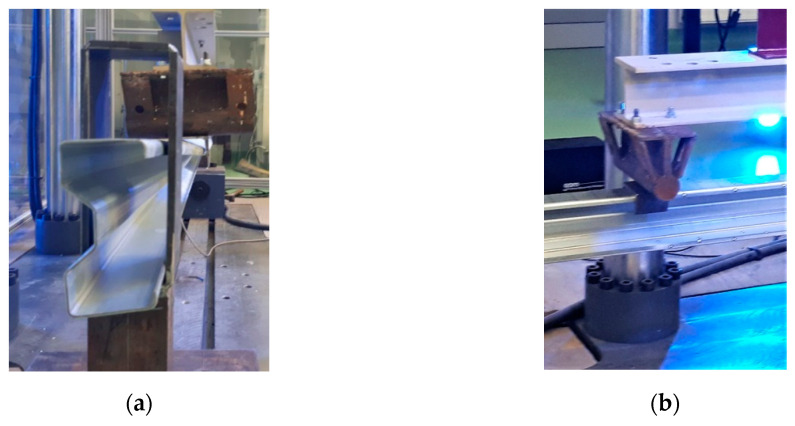
Details of the laboratory stand: (**a**) beam support detail; (**b**) of the load application through the hot-rolled C100 profile.

All tests were carried out in a certified laboratory at Lublin University of Technology. The process of beams loading was carried out with a Zwick and Roel testing machine (ZwickRoell GmbH and Co. KG, Ulm, Germany). Figure 4a shows a photo of the hinge screw connection at the support of the test stand. Such a connection allows free rotation in the beam plane and thus it is possible to obtain the so-called fork support. Additionally, to prevent local damage of the analyzed beams at the points of application of the concentrated force from the testing machine, a hot-rolled steel C100 profile (100-mm long) was used. This allowed the load to be distributed over the entire width of the upper flange (Figure 4b). The process of loading the beams was controlled with an extendable piston press (speed 1 mm/min), registering the force every 0.01 s.

**Figure 5:**
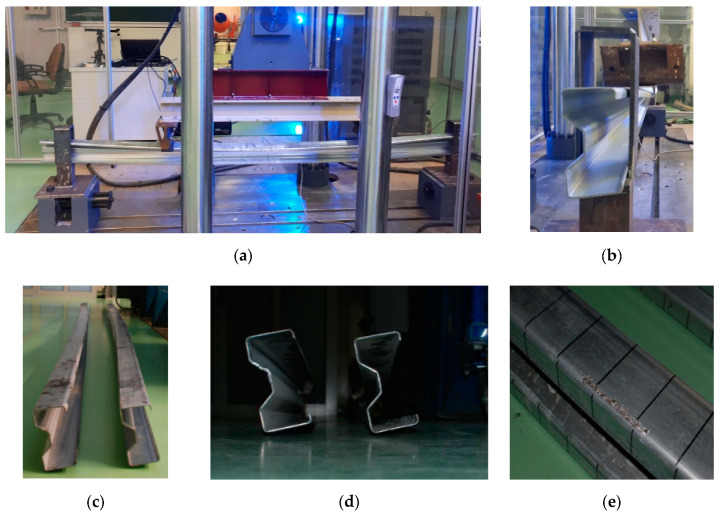
Failure mode of the beams of the third test stage: (**a**,**b**) beams in the loading phase, (**c**,**d**) beam failure modes, (**e**) local damage of the beam after the test scratch at the place of load application.

It should be pointed out that beams made of low cross-sections (Σ140 × 70 × 2.5), tested at this stage of study, are characterized by different structural behaviors than those observed in the case of high cross-section (Σ200 × 70 × 2) beams described in [1]. In the high beams [1], the deformation manifested by the opening of the beam’s cross-section and the debonding failure mode. A significant lifting of the upper beam flange was observed between the load application points. At the same time, the cross-section moved out of the vertical plane of the beam. In addition, local deformations of the upper flange were observed in the places where concentrated forces were applied. There was no damage to the lower flange. In [1], in all high beams the tape was debonded at a load of about 25 kN. Whereas, in case of 140-mm cross-section high beams, described in this part of the study, a totally different failure mechanism appeared. All the beams were characterized by a global form of destruction. The beams suffered both deflection and a significant rotation, so they were subject to failure in the form of lateral–torsional buckling. No local failure was observed. Only local scratches appeared on the top flange, where the load was applied. In one of the reinforced beams, the CFRP tape was partially subjected to debonding. The way the beam works and its deformation appearing at a load of 25 kN are presented in Figure 5.

**Figure 6:**
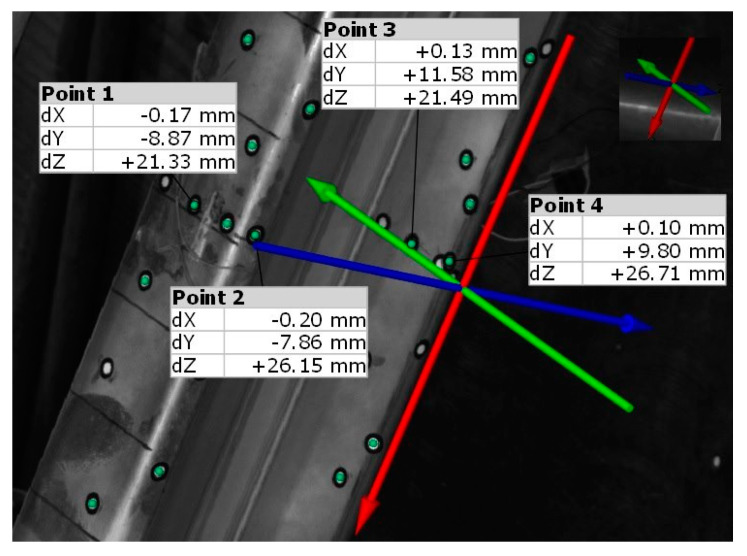
Displacements of individual points of cross-section in the middle of the span of one of the tested beams with a load of 25 kN.

Examples of the displacements of measuring points of the selected sample in the middle of its span obtained with a load of 25 kN are presented in Figure 6.

During the tests, the tape was observed to be debonded at the steel–glue surface in only one beam. The debonding occurred only at one side of the composite tape (CFRP) on ca. 10% of its length and appeared with a load exceeding 30 kN.

**Figure 7:**
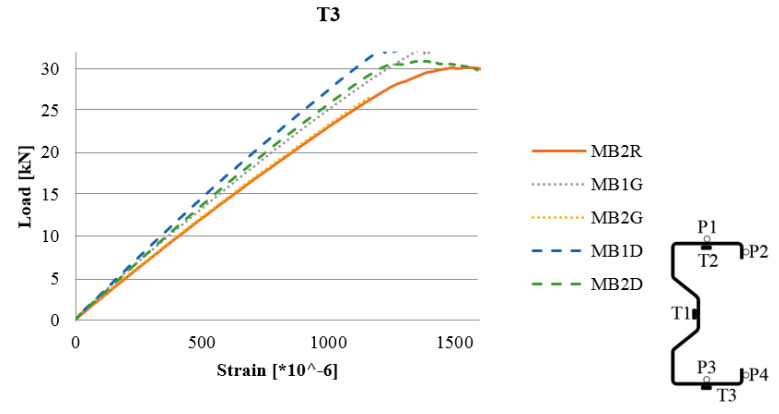
Load–strain diagram T3 strain gauge reading.

An example diagram of the load–strain relationship obtained from readings from an electrofusion strain gauge (TENMEX TFs-10, resistance 120 Ω ± 0.2%, Lodz, Poland) for different CFRP tape reinforcement locations is shown in Figure 7, respectively. The diagrams of the load–strain relationship based on the readings of the remaining strain gauges (T1 and T2) are shown in the further figures.

The value of the destructive force was adopted at the level of 25 kN. This assumption is due to the fact that up to this load level the correct readings were obtained for all strain gauges. Consequently, further analyses were carried out assuming that the limit load was equal to 25 kN. Moreover, at this load level, the effect of using CFRP tapes on reducing the displacements and strains of the tested sigma beams was described. Table 2 shows the strain values of each of the tested beams, obtained on the basis of readings from electrofusion strain gauges at a load level of 25 kN.

The percentage increase or reduction in the strain of each tested beam in relation to the strain determined for the reference beams was calculated based on the following formula:

(1) $$\rho_{\varepsilon i}=\left(\frac{\varepsilon_{i}}{\varepsilon_{\mathrm{ref}}}-1\right)*100\%$$

where:$\;\rho_{\varepsilon i}$—reduction or increase in strain of a given sample expressed as a percentage,$\;\varepsilon_{i}$—strain of a given sample, and $\varepsilon_{\mathrm{ref}}$—arithmetic mean value of the two reference beams strain (B1R and B2R).

**Figure 8:**
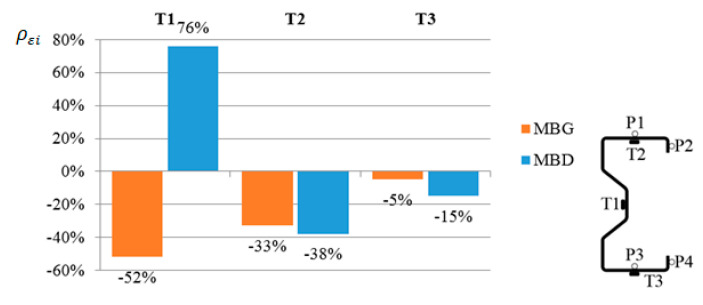
Strain percentage change in relation to the beams without reinforcement, read from a strain gauge at a load level of 25 kN and for different locations of the CFRP tape.

From the obtained results, the arithmetic mean value was determined for the beams reinforced in the upper flange (MBG) and reinforced in the bottom flange (MBD), which is presented in Figure 8.

**Figure 9:**
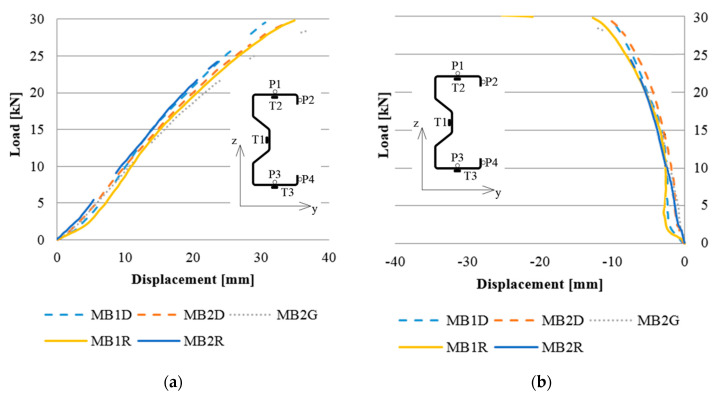
Load–displacement correlation measured at point P2 in the (**a**) vertical direction (z), (**b**) horizontal direction (y).

As already mentioned, a measurement of vertical and horizontal displacements at four selected points was performed using the Aramis system. An example of a load–displacement diagram is shown in the Figure 9.

Then, using the Formula (2), the displacement percentage change in relation to the reference beams at a load of 25 kN and for different positions of the CFRP reinforcement was determined.

(2) $$\rho_{ui}=\left(\frac{u_{i}}{u_{\mathrm{ref}}}-1\right)*100\%$$

where:$\;\rho_{ui}$—percent change in displacement of the *i*th sample,$\;u_{i}$—displacement of the *i*th sample, and $u_{\mathrm{ref}}$—displacement of reference beam.

**Figure 10:**
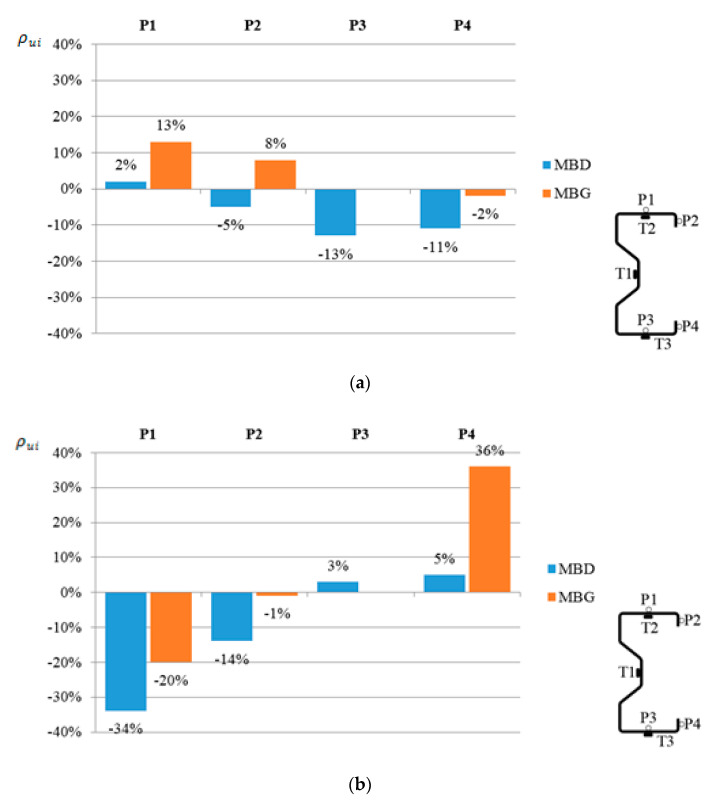
Percentage change in the displacement in relation to beams without reinforcement, read from points P1, P2, P3, at a load level of 25 kN, for different CFRP tape positions: (**a**) vertical displacement; (**b**) horizontal displacement.

The percent change in displacement is shown in Figure 10. Figure 10a refers to the changes in the vertical displacement, while Figure 10b is related to the horizontal displacement change. In the figures presented, the increase in displacement is represented by positive values, while negative values show a decrease in displacement. Note that the given values of displacement are the arithmetic mean value determined for the beams reinforced in the upper flange (MBG) and reinforced in the bottom flange (MBD).

It can be noticed that in the case of low beams subjected to significant rotation, bonding of the CFRP tape to the upper (compressed) flange contributed to the reduction in horizontal displacements to a lesser extent compared to the case of placing CFRP on the bottom (tensioned) flange. Moreover, in the case of displacements in the vertical direction it even contributed to the increase in displacement. This is surprising, because in the case of high beams [1], gluing the tape to the upper flange allowed for a significant limitation of horizontal displacements. In the case of the analyzed, i.e., low and slender beams, it can be stated that placing the CFRP tape on the upper flange is more advantageous when the aim is to limit the strain of the beams.

## 3. Numerical Model

The model of thin-walled steel beams made of the sigma-type cross-section (Σ140 × 70 × 2.5 profile) was made with the use of shell finite elements (FEs) with a linear shape function. The washers used to transfer the load directly from the press to the beam and the support clamps (whose shape reflected the fork support), similarly to the tests described in the paper [1], were modelled as non-deformable shell elements.

Both clamps and washers were assigned reference points in which the necessary boundary conditions were directly defined. Reference points have already been defined at the design stage of these parts (for washers these points are placed at the center of gravity, and support clamps are placed at the actual joints). The load was realized by applying two forces corresponding to the direction of the *Y* axis (each of the forces equal to 16,000 N), at reference points associated with non-deformable washers.

The numerical model takes into account the properties of the contact interaction in the normal direction (using Hard Contact) and tangential (without friction). In order to properly reproduce the experimental studies, contact interactions were introduced between the steel beam and support clamps and the beam and washers (used to transfer the load). The numerical model consisted of 16,104 nodes with a number of FEs equal to 15,587 (of which 14,147 were S4R-type elements, i.e., deformable shell elements), while 1440 were R3D4-type elements, i.e., non-deformable linear shell elements, from which support clamps and washer components were prepared. The material behavior was described by means of an elasto-plastic model with the bilinear characteristics [24,25]. In the case of reinforced beams, CFRP tapes (made of orthotropic material [26,27]) were modelled as shell FEs connected directly to the beam using TIE relations. The material properties used in numerical calculations are presented in Table 3.

**Figure 11:**
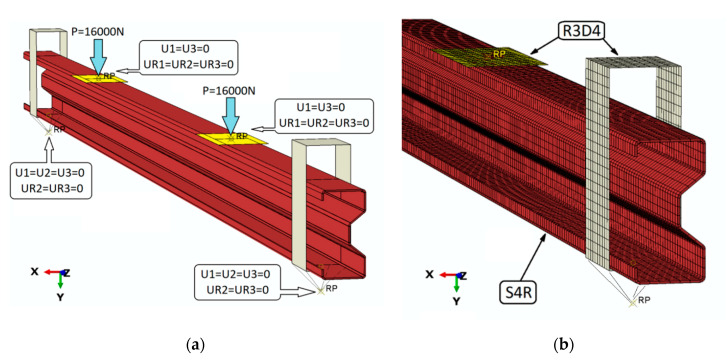
Numerical model for the third stage of study: (**a**) boundary conditions, and (**b**) mesh presentation.

The boundary conditions of the numerical model are shown in Figure 11.

## 4. FEM Results 

During the numerical analysis, the strains of the beam were measured in places corresponding to the actual location of T1, T2 and T3 electrical resistance strain gauges used directly during laboratory tests. The strain from the Abaqus program was read as Max. In-Plane Principal (Abs). 

**Figure 12:**
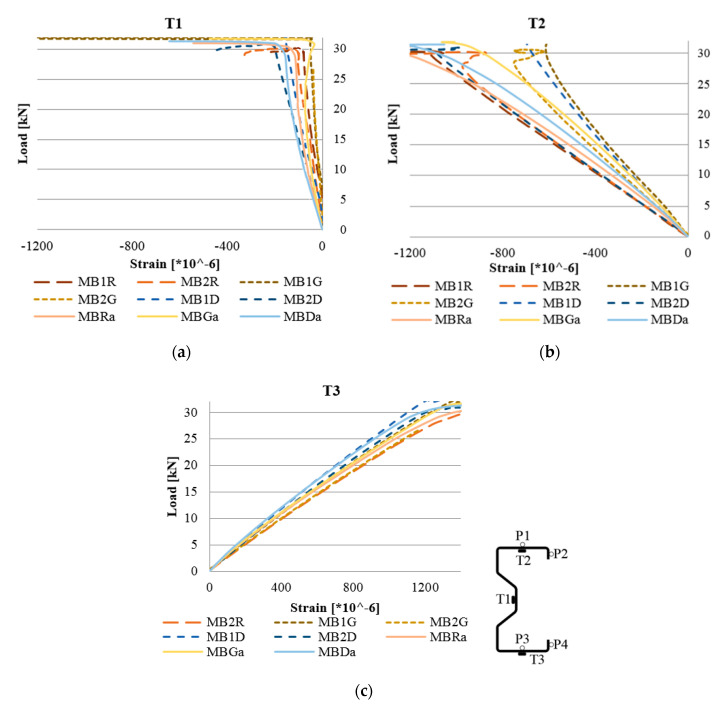
Load–strain relationship obtained from electrical resistance strain gauges in a laboratory test and at similar points in the finite element (FE) model: (**a**) in place of a strain gauge T1, (**b**) in place of a strain gauge T2, and (**c**) in place of a strain gauge T3.

In numerical analyses, the beam without reinforcements was marked as MBRa, while the reinforced beam in the upper flange was marked as MBGa and the beam reinforced with CFRP tape in the bottom flange was marked as MBDa. Comparison of laboratory and numerical results are shown in Figure 12.

**Figure 13:**
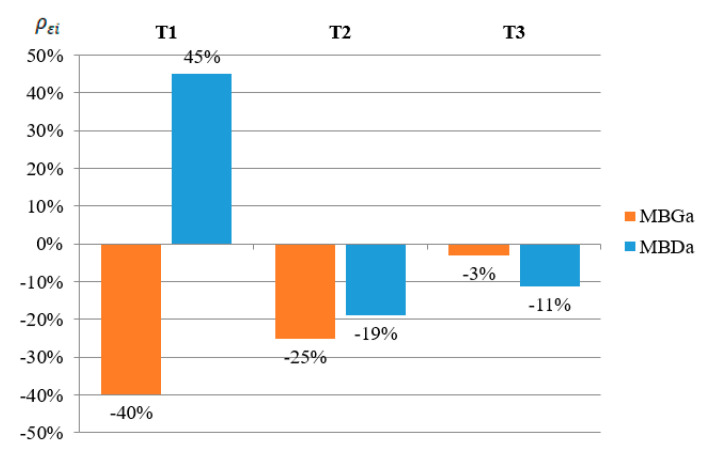
Strain percentage change in relation to the beams without reinforcement, read at T1, T2 and T3 at a load level of 25 kN and for different locations of the CFRP tape.

Figure 13 shows a graph similar to Figure 8. Based on the results obtained from the numerical analysis, it can be concluded that in the case of points T1 and T3 a good agreement with the results of laboratory tests was obtained. In the case of reading at the point of the T2 strain gauge, the results of numerical analyses differ from the results of laboratory tests. In laboratory tests, placing the CFRP tape on the bottom flange allowed for the greatest reduction in deformations in the top flange, while in the case of numerical analyses the situation was the opposite. This is due to the fact that the laboratory tests were carried out on several beams, which showed a certain dispersion of the results, while the data presented in Figure 8 are the arithmetic mean of the obtained results.

**Figure 14:**
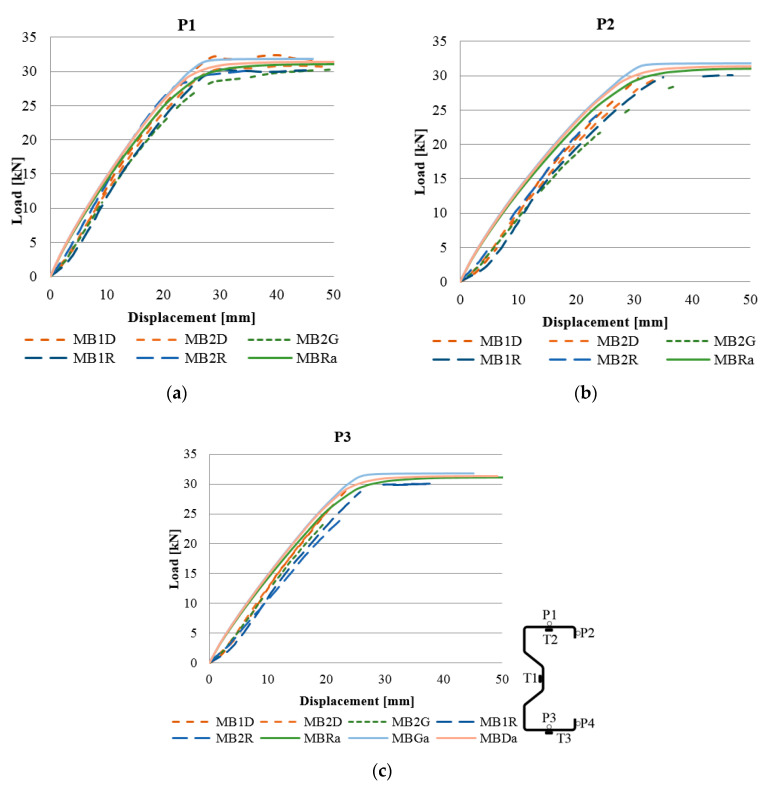
Load–displacement relationship in the vertical direction during laboratory tests and at the corresponding point in the FE model, for points: (**a**) P1, (**b**) P2, and (**c**) P3.

During the laboratory tests, displacements of samples were controlled at four points, marked P1, P2, P3 and P4. Figure 14 presents the results of vertical displacements for selected points to check the compatibility of numerical analyses and laboratory tests.

**Figure 15:**
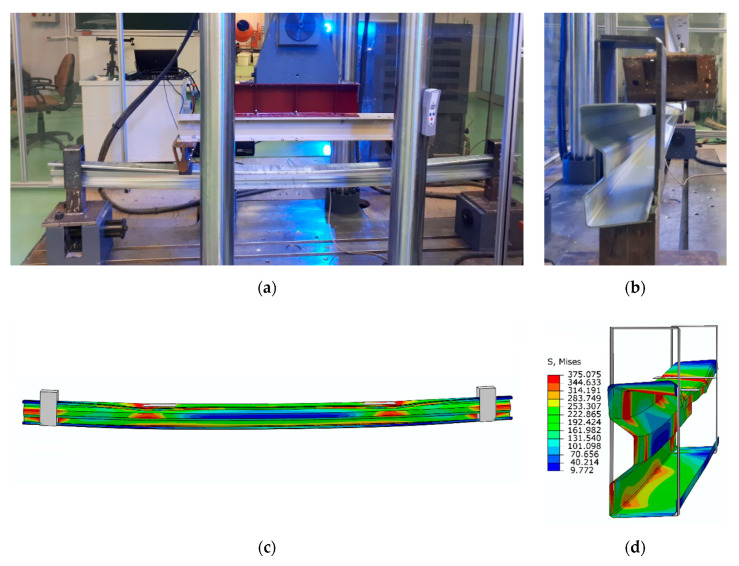
Comparison of beam Mises strains during laboratory tests and numerical analyses: (**a**) EXP (front side), (**b**) EXP (cross-section), (**c**) FEM (front side), (**d**) FEM (cross-section).

Analyzing the graphs presented in the above figures, concerning the strains obtained for the beams, it should be stated that the numerical models, in terms of quality, represent the actual response of structures reinforced with CFRP tapes. In other words, they represent the same trend in terms of both laboratory test results and numerical analyses. In quantitative aspects, the strain readings from electric resistance strain gauges for the beams of a given group (reference, reinforced in the lower and upper flange) slightly differed. At a level of load of 25 kN, the difference between the results obtained reached a maximum of 12% and an average of 3.5–5.5% for strain gauges T2 and T3. In the case of displacement, the results of laboratory tests and numerical analyses, presented in Figure 14, are characterized by a similar character of the load–displacement equivalence path. The discrepancy between the arithmetic mean value of the displacement, obtained for beams with the same reinforcement method, and the corresponding FEM numerical model, in none of the analyzed cases, exceeded 13%. In addition, the form of beam deformation obtained on the basis of numerical and laboratory tests (Figure 15) is compatible, which additionally confirms the proper method of modelling the tested thin-walled sigma beams.

## 5. Results and Discussion

The following paper presents a method of retrofitting steel thin-walled sigma beams with CFRP tapes. As a result of the conducted research and analyses, satisfactory results were obtained, showing the effectiveness of this type of reinforcement. A good agreement was obtained between the finite element method analysis and the experimental studies.

The 140-mm high cold-formed sigma steel beams described in this article were characterized by a smaller cross-section wall slenderness and greater member slenderness ratio, referring to those tested in [1]; therefore, global forms of deformation were observed. The beams described in [1] (higher and with a thinner cross-sectional wall) were characterized by a greater cross-section wall slenderness and a smaller member slenderness ratio, so they were characterized by local forms of deformation. The beneficial effects of reinforcing with tapes were obtained for both high and low cross-section beams—i.e., for elements with different slenderness ratios.

The authors of this study admit that in the case of cold-formed elements, due to the small cross-sectional area, the reinforcement with CPRF tapes may itself increase the cross-sectional geometrical characteristic of the element and thus increases its bearing capacity. It is worth noting, however, that as a result of the reinforcement of the beams with a tape located at the bottom flange of the steel beam, a 22% increase in the moment of inertia about the *y* axis, 8% increase about the *z* axis and a change in the bending section modulus with respect to the *z* axis by 5% were obtained.

It should be noted that by gluing the CFRP tape to the bottom flange, it is possible to reduce the vertical beam displacement by 13% and the horizontal beam displacement by 34%. It should therefore be emphasized that the benefits of reinforcing the beam cross-section using CFRP tapes are much greater than would result from only the change of the geometrical characteristics of its cross-section. This confirms the high efficiency of reinforcing thin-walled steel sections with CFRP tapes. As it was already mentioned in [1], attention has to be drawn to the fact that the proposed method differs from the classic approach to reinforcing steel structures.

The proposed method deliberately deviates from the principle of convergence of the centers of gravity of both the beam cross-section and CFRP reinforcement. This is dictated both by the fact that the sigma-type section has only one symmetry axis, as well as due to the limitations related to the access to the element during the reinforcement process “in situ”.

In addition, when designing the reinforcement of steel structure elements with tapes, various possible forms of failure should be taken into account, such as adhesive debonding along the surface of the steel–adhesive or composite–adhesive interface, CFRP delamination, and cohesive failure in the adhesive layer. This is due to the fact that the steel strength is higher than that of traditional adhesives used to strengthen structures [22].

In the presented stage of the research, as well as in the tests described in [1], a constant thickness of the adhesive layer was used, equal to 1.3 mm. It should be noticed that in [1], the tape was detached at the steel–glue interface in each of the tested samples. In the tests described in this work, debonding occurred in only one sample at a high level of load. This may indicate that the debonding of the tapes was related to the local deformation of the beams. The authors are still unable to indicate the relationship between the adhesive layer thickness and the value of load at which the tape detaches from the tested beams. The influence of the glue layer thickness on the effectiveness of reinforcement using CFRP tapes will constitute the next stage of laboratory research. 

Due to the many possible forms of failure as well as the limited knowledge of the behavior of CFRP tapes glued to steel, it will be advisable to conduct further research before implementing the proposed method in engineering practice.

The aim of the study was to answer the question posed by the industry on the possibility of reinforcing steel cold-formed roof purlins made of sigma-type profiles to be used in steel halls. The conducted tests were of a pilot nature and were aimed at demonstrating the effect of the location of CFRP tapes on steel sigma beams in laboratory conditions. The obtained results do not refer directly to the reinforcement of the existing structure—for example, by the fact that it would have to be completely unloaded and devoid of cooperation with other elements. The satisfactory results obtained in the research concerning the limitation of displacement and strain of the tested beams strengthened with the CFRP tape are the motivation for further research on the reinforcement of roof purlins.

## 6. Conclusions

The obtained results of laboratory tests and numerical analyses allow us to state that the reinforcement in the form of CFRP tapes has a positive effect on the displacement and reduction in deformations of thin-walled cold-formed steel beams made of Σ140 × 70 × 2.5 profiles. Based on the performed analyses, detailed conclusions were formulated:

- Placing the composite tape on the bottom flange of the beam allowed to reduce the strain in the bottom flange by 15%.
- By sticking a composite tape (CFRP) to the top (compressed) flange, it is possible to reduce the average strain on the top flange (33%) and in web (52%).
- Placing the composite tape on the bottom flange reduced the horizontal displacement perpendicular to the longitudinal axis of the beam on the top flange by 34%.
- Placing of the composite tape on the bottom flange allowed to reduce the vertical displacement of the beam by 13%.

Summing up, it should be noted that reinforcement with CFRP tape adhered to the upper flange can be very advantageous for beams subjected to high rotation, when the aim is to limit the strains of the beams. The innovative approach presented in the paper, which consists of placing the tape on the inside of the beam flange, allows to remove the technological limitations of reinforcing beams during exploitation. Moreover, the authors, as in [1], performed displacement measurements using an innovative method based on the use of the Tritop system in combination with two Aramis system devices, which recorded the behavior of the tested beam on both sides. This method allows for a reliable measurement of the displacements of points subject to large displacements, which is impossible in the case of traditional methods—e.g., when using inductive displacement gauges. Overall, it can be concluded that the traditional approach to the strengthening of steel beams, according to which it is recommended to place CFRP tapes at the bottom flange, cannot be considered as a universal method. In the case of low beams, which undergo global deformation during bending, the placement of CFRP tapes at the tensioned flange allowed the limiting of displacements to the greatest extent, but it did not prove to be the best solution in the case of the need to limit the deformations of the tested beams.

## Figures and Tables

**Table 1:** The geometrical characteristics of Σ140 × 70 × 2.5 cross-section.

Weight	FA	e_yy_	e_zz_	J_y_	J_z_	W_y_	W_z_	i_y_	i_z_
(kN/m)	(cm^2^)	(cm)	(cm)	(cm^4^)	(cm^4^)	(cm^3^)	(cm^3^)	(cm)	(cm)
6.13	7.81	2.613	6.95	223.00	38.80	32.09	14.85	5.34	2.23

**Table 2:** The strain of individual samples at the load level of 25 kN.

	Strain (×10^−6^)		
	T1	T2	T3
MB1R	–70.3	–949.8	
MB2R	–95.1	–909.0	1097.7
MB1G	–42.7	–549.4	994.8
MB2G	–37.0	–688.8	1082.5
MB1D	–124.9	–578.8	900.2
MB2D	–166.9		966.1

**Table 3:** Material properties of Sigma Beam and CFRP.

Elastic-Plastic Model (Sigma Beam)		Elastic Model (CFRP)	
Young Modulus E (MPa)	201,797	Young Modulus E_1_ (MPa)	142,010
Poisson Ratio (-)	0.282	Young Modulus E_2_ (MPa)	8010
Yield Point (MPa)	418.5	Poisson Ratio (-)	0.308
Tensile Point (MPa)	473.56	Kirchhoff Modulus G_12_, G_23_, G_13_ (MPa)	4501
Elongation at break (A%)	16		
